# Professional burnout, stress and job satisfaction of nursing staff at a university hospital^1^

**DOI:** 10.1590/0104-1169.0284.2586

**Published:** 2015-07-03

**Authors:** Silvia Portero de la Cruz, Manuel Vaquero Abellán

**Affiliations:** 2 MSc, Researcher, Departamento de Enfermería, Facultad de Enfermería, Universidad de Córdoba, Córdoba, Spain; 3 PhD, Professor, Facultad de Enfermería, Universidad de Córdoba, Córdoba, Spain

**Keywords:** Burnout, Stress, Job Satisfaction, Occupational Health

## Abstract

**OBJECTIVES:**

to describe the social and work characteristics of the nursing staff at a tertiary hospital in the Public Health Service of Andalucía, to assess the degree of professional professional burnout and job satisfaction of those professionals and to study the possible relation between the professional burnout variables and the stress and job satisfaction levels on the one hand and social and employment variables on the other.

**METHOD:**

descriptive and cross-sectional study in a sample of 258 baccalaureate and auxiliary nurses. As research instruments, an original and specific questionnaire was used to collect social and employment variables, the Maslach Burnout Inventory, the Nursing Stress Scale and the Font-Roja questionnaire. Descriptive, inferential statistics and multivariate analysis were applied.

**RESULTS:**

average scores were found for professional stress and satisfaction, corresponding to 44,23 and 65,46 points, respectively. As regards professional burnout, an average score was found on the emotional exhaustion subscale; a high score for depersonalization and a low score for professional accomplishment. Studies are needed to identify the scores on these subscales in health organizations and to produce knowledge on their interrelations.

## Introduction

As a consequences of the changes in the organizations and of the current globalization processes, the exposure to psychosocial factors in the professional sphere has been more frequent and intense^1^. When these are adverse to the development of the professional activity and the individual's quality of life, they turn into a higher level of stress for the professional^2^.

In the last two decades, there has been a growing concern with the effects of stress on the nursing professionals^3^, which represent the most numerous group of health professionals who deliver care to patients 24 hours per day^4^. According to the Occupational Health and Safety Survey of the American Nurses Association (ANA), the main concern for the nursing staff with regard to health and safety in the work environment is the acute or chronic effect of stress^5^. The work conditions in nursing imply the exposure to pain and death, interpersonal conflicts^6^, lack of autonomy and authority for decision making and the lack of definition of the professional role, which produce a state of chronic stress^7^. The individual response to these situations can be psychological, including symptoms like anxiety, irritation and depression, or psychosomatic, involving headaches, nausea and sleep problems, with possible negative impacts for patient safety and for the quality of care^8^.

The prolonged exposure to professional stress is associated with the professional burnout syndrome^9^, characterized by high levels of emotional exhaustion, which refers to the decrease or loss of emotional resources, the depersonalization or development of negative attitudes towards patients and, finally, the lack of personal accomplishments, which provokes trends to assess one's work negatively^10^. The consequences of professional burnout include mental fatigue, lack of motivation^11^, increased risk of cardiovascular diseases, musculoskeletal problems^12^, low output levels, low productivity and absenteeism^13^. 

Current evidence reveals job satisfaction as a predictor of the length of stay in a job, motivation and job productivity. The level of job satisfaction among nursing staff is dropping all over the world though^14^. The main sources of nursing dissatisfaction include lack of staff, high care pressure and lack of professional acknowledgement^15^.

The objectives in this study were to describe the social and employment characteristics of the nursing staff at a tertiary hospital in the Public Health System of Andalucía, to assess these professional's degree of work-related stress, professional burnout and job satisfaction and to study the possible relation between the professional burnout dimensions and the work-related stress and job satisfaction levels with social and employment variables.

## Methods

An observational, descriptive and cross-sectional study was undertaken at a tertiary hospital in the Public Health System of Andalucía. The data were collected between February and June 2014. The study subjects were nurses or auxiliary nurses who worked at the hospital, who had worked at the service where they were active in care for more than one year and who were active at the time of the data collection. Accepting an alpha risk of 0.05, for an absolute precision level of 5% in a bilateral contrast and an estimated prevalence of professional burnout corresponding to 25%^16^, a sample of 258 subjects was determined, assuming a population of 2242 subjects. The sample size was calculated in the software Epidat version 4.0, departing from the least advantageous prevalence. The researchers received data on the total resources allocated to the nursing staff from the nursing head of the hospital. The following exclusion criteria were adopted: being a nursing student/auxiliary nurse or being a professional in specialized educational training. 

The professionals were selected through random cluster sampling. The clusters corresponded to the services where the professionals worked. The hospital consists of 45 services. Using tables with random figures, eight services were selected in function of the sample size and the total number of nurses and auxiliary nurses working at the service. To foresee possible losses, two additional services were selected. All nursing professionals and auxiliary nurses from each of the selected services were considered for the study. 

The study received approval from the hospital's Institutional Review Board (protocol 227, reference 2491). A dossier was designed to collect information, including an information letter in which the voluntary and anonymous nature was highlighted, an explicit request for cooperation which the professionals signed and data to agree to participate in the study, an original and specific form that collected social and employment variables, the Nursing Stress Scale (NSS)^17^, the Maslach Burnout Inventory (MBI)^18^ and the Font-Roja questionnaire^19^. The researchers personally gave the dossier to the professionals at the start of each work shift (morning and afternoon). In addition, the service supervisors received the number of dossiers needed for the workers who were not present at the place of work at the time of the distribution because they were working night shifts. Completed dossiers were returned directly to the researchers at the end of the shift or to the service supervisors, for them to pass them on to the researchers. 

The *social and employment variables*, collected through an original and specific questionnaire, were: sex (woman, man), age (years), professional category (baccalaureate nurse, auxiliary nurse), marital status (single, married, separated-divorced, widowed), children (yes/no), relatives living at home (none, first-degree relative, second-degree relative, both), complementary education (yes/no), professional activity and/or complementary education beyond work journey (yes/no), type of contract (statutory, undefined, hired), trainees in the previous month (yes/no), weekly work journey, number of patients the professional is in charge of per day, professional experience (years), time on the current job (years). To measure the *work-related stress *in the nursing staff, the NSS validated in Spain was used. This scale consists of 34 items on potentially stressful situations. The possible response categories for each of the items are: never (0), sometimes (1), frequently (2), very frequently (3). This scale has a factorial structure and includes seven subscales: death and suffering (5 items), problems with doctors (5 items), insufficient preparation (6 items), lack of support (3 items), problems with other nursing team members (5 items), work burden (6 items) and uncertainty in treatment (4 items). The total score is equivalent to the simple sum of the items in each subscale divided by the number of items. Any mean score higher than 1 was considered as a nursing stress factor. The global work-related stress was obtained by adding up the scores of the 34 items, so that the global score ranges between 34 and 102 points^16^. In this study, the internal consistency of the global scale, represented by Cronbach's alpha coefficient, corresponded to 0.87. To measure the *degree of professional burnout*, the Spanish version of the MBI was sued. This questionnaire consists of 22 items. The participants answer according to how frequently they experience the feelings, ranging from never (0) to few times per year or less (1), once per month or less (2), few times per month (3), once per week (4), few times per week (5), every day (6). No valid cut-off points have been described in the literature for the clinical level in order to measure the existence of professional burnout and to be able to distinguish the cases. Nevertheless, high scores for emotional exhaustion and depersonalization and low scores for professional accomplishment suggest the presence of this syndrome. The sum of the scores determines three dimensions of professional exhaustion: emotional fatigue (9 items), depersonalization (5 items) and personal accomplishment (8 items). To interpret the scores obtained on the three scales, the following cut-off points were used: for emotional exhaustion, the scores range between 15 and 24 (<15 low, 15-24 medium and >24 high), for depersonalization between 4 and 9 (<4 low, 4-9 medium and >9 high) and, for personal accomplishment, between 33 and 39 (<33 high, 33-39 medium and >39 low)^17^. The Cronbach's alpha coefficient for the emotional exhaustion scale corresponded to 0.92, for depersonalization to 0.83 and for personal accomplishment to 0.82. *Job satisfaction *was measured using the Font-Roja questionnaire, validated for use in Spain. The questionnaire consists of 24 items. The participants answer according to the degree of agreement with different situations related to their job environment. The possible response categories for each of the items are: strongly disagree (1), disagree (2), neither agree nor disagree (3), agree (4), strongly agree (5). The items are grouped in 9 factor that permit exploring different dimensions that intervene in job satisfaction: satisfaction with work (degree of satisfaction the individual perceives conditioned by the job function, 4 items), work-related tension (degree of tension the work causes in the subject and which is manifested as fatigue, stress and professional burnout, 4 items), professional competency (extent to which the individual believes (s)he is prepared to perform daily work, 3 items), pressure at work (extent to which the individual perceives the work as a burden, 2 items), professional promotion (extent to which the worker believes (s)he can improve professionally and in terms of professional acknowledgement, 3 items), interpersonal relationship with heads (degree of satisfaction the social relationships with the heads provoke in the individual, 2 items), interpersonal relationship with peers (degree of satisfaction the social relationships with the peers provoke in the individual, 1 item), extrinsic characteristics of status (extent to which the individual believes the work offers fair remuneration and a level of independence in the organization and performance of the function, 2 items), and job monotony (extent to which the job routine affects the subject, 2 items). The total score corresponds to the simple sum of the items, divided by the number of items. Score 3 was considered as the median satisfaction level. The global or total job satisfaction was calculated by adding up the scores for the 24 items, ranging between 24 and 120 points^18^. The internal consistency of the global scale, represented by Cronbach's alpha coefficient, corresponded to 0.83.

The qualitative variables were expressed using absolute and percentage frequencies and the quantitative variables though means and standard errors. To compare the means between two independent groups, Student's t-test was applied, after confirming the normal distribution of the variables. 

For the comparison of means between more than two independent groups, variance analysis (ANOVA) was applied if the variables followed a normal distribution or Kruskal-Wallis' non-parametric test in case of a non-normal distribution. For p-values < 0.05, the two distinct groups were compared using Dunn's method, when Kruskal-Wallis non-parametric test was applied or Tukey, Scheffé or Bonferroni's method for variance analysis in function of the equality or not of the variances and sample size of the groups. To correlate the variables, Pearson's linear correlation coefficient was used, after proving the normal distribution of the variables. To estimate the variables related to professional burnout, three multiple linear regression models were developed, using the reverse variable selection method. The dependent variables were emotional exhaustion, depersonalization and personal accomplishment. The independent variables were age, sex, professional category, marital status (recoded as: married-unmarried), children, relatives living at home, professional activity and/or education beyond work journey, type of contract, trainees under professional's responsibility in previous month, weekly work journey, number of patients the professional is in charge of per day, work experience, time on the current job, global work-related stress level and global job satisfaction level. Using Wald's statistics, variables with p≥ 0.15 were eliminated from the model one by one. The Box-Tidwell test was used to assess the scale of the continuing variables. Possible interactions among the variables were studied. Variables with significance superior to 0.05 were studied as possible confounding factors, considering them as such if the percentage of change of the coefficients was superior to 15%. To prove the normality of the residues, the Kolmogorov-Smirnov test was applied. It was considered that there were no collinearity problems among the independent variables if the variance inflation factor was lower or equal to 10. To diagnose extreme cases, the analysis of studentized residuals was used. The adjusted determination coefficient R^2 ^was used to assess the goodness of fit. P-values inferior to 0.05 were considered significant in all statistical tests.

For the statistical analysis, the software G-Stat version 2 was used.

## Results

In total, 258 properly completed dossiers were received. In the study sample, 77.52% of the professionals were women. The mean age was 49.21 (32 -65) years. 64.34% held a nursing degree. On average, the professionals attended to 7.90±5.21 patients per day. 69.38% were married. In addition, 212 professionals had children. On average, the professionals worked 36.18±6.98 hours per week. 73.26% lived with first-degree relatives. The mean professional experience was 24.16±8.19 years. 72.09% of the professionals worked on a statutory contract. The mean time on the job was 10.43±8.39 years. 50.78% of the professionals were in charge of trainees. As observed in Table 1, the mean work-related stress score was 44.23±12.97, ranging between 13 and 76 points. The minimum score for emotional exhaustion was 0 and the maximum 49; scores for depersonalization ranged between 0 and 25 points; and, for personal accomplishment, between 10 and 48 points. For the mean global satisfaction, the minimum score was 51 points and the maximum 85.

**Table 1.** Description of dimensions and/or subscales of the Nursing Stress Scale, Maslach Burnout Inventory and Font-Roja questionnaire in nurses and auxiliary nurses. Andalucía, Spain, 2014

		Arithmetic mean (Standard error) n=258	95% confidence interval
Nursing Stress Scale			
	Total work-related stress (points)	44.23 (12.97)	42.64 – 45.82
	Work burden (points)	1.57 (0.49)	1.51 – 1.63
	Death and suffering (points)	1.46 (0.66)	1.38 – 1.54
	Insufficient preparation (points)	1.37 (0.74)	1.28 – 1.46
	Lack of support (points)	1.39 (0.66)	1.31 – 1.47
	Uncertainty in treatment (points)	1.10 (0.20)	1.08 – 1.12
	Problems with medical staff (points)	1.02(0.58)	0.95 – 1.09
Maslach Burnout Inventory			
	Emotional exhaustion (points)	17.48 (11.93)	16.02 – 18.94
	Depersonalization (points)	9.04 (5.53)	8.36 – 9.72
	Personal accomplishment (points)	39.22 (8)	38.24 – 40.20
Font-Roja			
	Global mean satisfaction	65.46 (7.04)	64.60 – 66.32
	Work-related tension (points)	2.41 (0.71)	2.32 – 2.50
	Professional competency (points)	2.37 (0.68)	2.29 – 2.45
	Work pressure (points)	2.78 (1.03)	2.65 – 2.91
	Professional promotion (points)	2.77 (0.48)	2.71 – 2.83
	Interpersonal relationship with heads (points)	3.84 (0.66)	3.72 – 3.92
	Interpersonal relationship with work colleagues (points)	3.85 (0.84)	3.75 – 3.95
	Extrinsic characteristics of status (points)	2.05 (0.72)	1.96 – 2.14
	Job monotony (points)	2.86 (0.72)	2.77 – 2.95

The level of emotional exhaustion was 4.74 points higher among professionals who did not supervise trainees when compared to supervisors (p<0.01). In the population, adopting a 95% confidence level, this score ranged between 1.87 and 7.61 points. As regards personal accomplishment, the professionals who lived with first-degree relatives obtained a mean score significantly higher by 9.83 points than professionals who lived with second-degree relatives. In the population, with a 95% confidence level, this score ranged between 3.92 and 15.74 points (Table 2).

**Table 2.** Association and correlation between professional burnout dimensions and social and employment variables in nurses and/or auxiliary nurses. Andalucía, Spain, 2014

		EE*(points) n=258		p^||^	DP^†^(points) n=258	p^||^	PA^‡^(points) n=258	p^||^
		Mean(SE)^§^			Mean (SE)^§^		Mean (DT)^§^	
Sex								
	Man	18.24(12.25)		0.58	9.78(5.67)	0.25	39.34(7.90)	0.90
	Woman	17.25(11.85)			8.83(5.48)		39.19(8.05)	
Professional category								
	Nurse	16.92(11.74)		0.31	8.84(5.79)	0.42	39.43(7.72)	0.59
	Auxiliary nurse	18.49(12.25)			9.41(5.03)		38.86(8.52)	
Estado civil								
	Single	20.60(11.63)		0.08	11.83(4.29)^a^	<0.001^¶^	40.09(7.20)	0.14
	Married	17.40(12.02)			9.17(5.36)^a^		39.59(7.96)	
	Separated	13.06(10.28)			6.39(5.85)		37.22(8.97)	
	Widowed	25.37(12.02)			6(6.82)		36.38(7.07)	
Children								
	Yes	17.06(12.14)		0.24	8.86(5.70)	0.26	38.88(8.32)	0.13
	No	19.37(10.83)			9.87(4.61)		40.83(6.19)	
Living with relatives								
	No	19.62(11.41)		0.11	10.08(4.83)	0.24	40.10(6.13)^b^	<0.01^¶^
	First-degree relatives	16.79(11.94)			8.85(5.68)		39.68(7.95)^b^	
	Second-degree relatives	23.69(13.60)			10.54(5.53)		29.85(9.97)^c^	
	Both	15.41(10.41)			7.71(5.16)		39.35(6.80)	
Complementary education								
	Yes	18.47(12.39)		0.22	9.07(5.73)	0.95	39.31(7.68)	0.87
	No	16.63(11.49)			9.02(5.38)		39.15(8.30)	
Extra job								
	Yes	17.71(12.04)		0.80	9.49(5.21)	0.31	39.13(8.30)	0.89
	No	17.33(11.89)			8.77(5.71)		39.28(7.86)	
Type of contract								
	Statutory	17.41(11.73)		0.94	8.78(5.66)	0.37	39.13(8.17)	0.96
	Undefined	18.33(13.25)			9.80(5.31)		39.42(7.87)	
	Hired	16.52(11.27)			9.56(4.98)		39.52(7.31)	
Trainees								
	Yes	15.15(11.67)		<0.01	7.94(5.73)	0.001	39.21(7.75)	0.98
	No	19.89(11.75)			10.18(5.10)		39.24(8.29)	
		Coef. Corr. Pearson (r)	R^2^**		Coef. Corr. Pearson (r)	R^2^**	Coef. Corr. Pearson (r)	R^2^**
Age (years)		0.02	0.02%		-0.02	0.05%	-0.07	0.48%
Hours work/week		0.04	0.13%		0.04	0.15%	-0.005	0.03%
Number of patients professional is in charge of daily		-0.03	0.12%		-0.05	0.21%	0.04	0.17%
Work experience (years)		0.02	0.04%		-0.05	0.21%	-0.03	0.08%
Time on the job (years)		-0.002	0.04%		0.04	0.20%	-0.04	0.19%
EE*		1						
DP^†^		0.67^††^	46.11%		1			
PA^‡^		-0.40^††^	15.64%		-0.32^††^	10.16%	1	
Global work-related stress		0.29^††^	8.57%		0.33^††^	10.86%	-0.05	0.29%
Global job satisfaction		-0.11	1.26%		-0.05	0.26%	0.06	0.31%

*Emotional exhaustion †Depersonalization ‡Personal accomplishment §Standard error ||p: Significance level ¶Significance obtained using the Kruskall-Wallis test and Dunn's method for a posteriori comparisons: a= Significant difference from separated-divorced, b= Significant difference from second-degree relatives, c= Significant difference from first and second-degree relatives. **Adjusted determination coefficient ††p value < 0.001 Regression lines: emotional exhaustion =4.23+1.46xdepersonalization, emotional exhaustion =5.57+0.27xglobal work-related stress, emotional exhaustion =40.5-0.59xpersonal accomplishment, depersonalization=3.54+0.31x emotional exhaustion, depersonalization=2.83+0.14xglobal work-related stress, depersonalization=17.68-0.22xpersonal accomplishment, personal accomplishment=43.40-0.46xdepersonalization, personal accomplishment=43.86- 0.27x emotional exhaustion.

As regards the global job satisfaction, a positive and significant relation was found with the professionals' age: for each additional year of age, the job satisfaction increases by 0.22 points (Table 3).

**Table 3.** Association and correlation between global work-related stress level and global satisfaction level in nurses and auxiliary nurses. Andalucía, Spain, 2014

		Global work-related stress (points) n=258	p^†^	Global job satisfaction (points) n=258	p^†^
		Mean (SE)*		Mean (SE)*	
Sex					
	Man	44.86(14.01)	0.67	65.86(6.92)	0.08
	Woman	44.04(12.68)		64.05(7.34)	
Professional category					
	Nurse	42.50(13.52)	0.08	65.03(6.63)	0.19
	Auxiliary nurse	47.35(11.32)		66.23(7.70)	
Marital status					
	Single	50.03(9.63)	0.07	64.82(6.22)	0.85
	Married	44.18(12.88)		65.65(7.28)	
	Separated	38.83(12.59)		64.86(6.69)	
	Widowed	44.25(19.88)		66.63(7.46)	
Children					
	Yes	43.94(13.23)	0.45	65.47(7.17)	0.94
	No	45.54(11.74)		65.39(6.47)	
Living with relatives					
	No	46.69(11.92)	0.59	65.90(6.70)	0.16
	First-degree relatives	43.85(13.17)		65.18(7.19)	
	Second-degree relatives	42.15(9.86)		64.15(6.32)	
	Both	44.41(15.22)		68.53(6.33)	
Complementary education					
	Yes	44.28(12.89)	0.96	64.67(6.88)	0.10
	No	44.19(13.09)		66.12(7.13)	
Extra job					
	Yes	43.61(13.21)	0.33	64.95(7.06)	0.37
	No	45.23(12.57)		65.77(7.03)	
Type of contract					
	Statutory	43.65(12.72)	0.38	65.83(7.20)	0.18
	Undefined	44.80(13.51)		65.31(6.71)	
	Hired	47.26(13.78)		63.15(6.22)	
Trainees					
	Yes	41.40(14)	<0.001	66.05(6.25)	0.17
	No	47.15(11.13)		64.84(7.75)	
		Pearson Corr.	R^2‡^	Pearson Corr.	R^2‡^
Age (years)		0.03	0.09%	0.22^§^	4.71%
Hours work/week		-0.06	0.38%	0.11	1.29%
Number of daily patients		0.04	0.13%	0.09	0.80%
Work experience (years)		0.01	0.02%	0.07	0.56%
Time on current job (years)		0.07	0.53%	0.16	2.47%
EE^||^		0.29^§^	8.57%	-0.11	1.26%
DP^¶^		0.33^§^	10.86%	-0.05	0.26%
PA**		-0.05	8.29%	0.06	0.31%
Global work-related stress		1		0.09	0.76%
Global job satisfaction		0.09	0.76%	1	

*Standard error †Significance level ‡Adjusted determination coefficient ||Emotional exhaustion ¶Depersonalization **Personal accomplishment §p-value <0.01 Regression line: global job satisfaction=54.86+0.22xage.

The conditions of the multiple linear regression models regarding normality of residuals, non collinearity between independent variables and non-existence of extreme values were complied with for the dimensions: emotional exhaustion, depersonalization and personal accomplishment. Like the rest of the variables, for each additional point in the global job satisfaction, the emotional exhaustion score dropped by 0.22 points (Table 4).

**Table 4.** Multiple linear regression model between burnout dimensions and social and employment characteristics of nurses and auxiliary nurses. Andalucía, Spain, 2014

Dependent variable	Independent variables	Regression coefficients	P*
EE^†^	Global job satisfaction	-0.22	0.03
	Trainees		
	Global work stress = 30 points	-6.84	
	Global work stress = 64 points	1.86	0.003
	Global work stress = 98 points	10.56	
DP^‡^	Trainees		
	Global work stress = 30 points	-8.17	
	Global work stress = 64 points	-7.30	0.002
	Global work stress = 98 points	-6.44	
PA^§^	Living alone (reference)	1	
	First-degree relatives	-0.43	0.76
	Second-degree relatives	-10.26	<0.001
	Both	-0.75	0.74

*Significance level †Emotional exhaustion ‡Depersonalization §Personal accomplishment Adjusted determination coefficient for emotional exhaustion = 12.36%. F= 10.06 (p<0.001). Adjusted determination coefficient for depersonalization = 15%. F=16.11 (p<0.001). Adjusted determination coefficient for personal accomplishment = 6.27%. F = 6.73 (p<0.001.

## Discussion

In this study, the sample was predominantly female. The mean age was 49.21 years. In addition, 69.38% were married. These results were similar to other studies in Spain^20^^-^ 21 and abroad^6^.

The mean scores on the professional burnout dimensions situated the sample at a medium level on the emotional exhaustion subscale; a high level for depersonalization and a low level for personal accomplishment. The comparison of the mean scores for the professional burnout dimensions with other studies^22^^-^ 23 proves that the mean scores in this study are similar for the emotional exhaustion and depersonalization dimensions. As regards personal accomplishment, the mean score was slightly higher in this study. 

A significant and positive relation was found between the professionals' global stress level and their emotional exhaustion and depersonalization. This result is in line with the findings of another study^11^. Nevertheless, no significant relation was found between the global stress level and personal accomplishment. 

In the adjusted analysis, the association between emotional exhaustion and the global mean satisfaction derives from the nursing professionals' feeling of emotional impact of their work and the work relations established at the heart of the organization, reducing their job satisfaction^23^.

The mean score for depersonalization was significantly higher among single and married than among separated or divorced professionals. The relation between professional burnout dimensions and the marital status is a frequent source of discussion. While some researchers describe that marriage hampers the presence of the syndrome in health professionals (taking the form of a less cold attitude towards the patients)^21^, others consider that marriage is not a variable that significantly affects the professional burnout dimensions, considering that the social support received from the partner causes this influence^20^.

As regards the personal accomplishment dimension and adjusted by the other variables, professionals who lived with second-degree professionals showed a 25-percent lower score for personal accomplishment when compared to professionals who lived alone. The professionals may not perceive sufficient support from their relatives, which influences the professional's good performance.

The study limitations basically derive from the cross-sectional design, which only permits the analysis of associations between the variables, without the possibility of establishing causal relationships. In addition, the veracity factor in the professionals' answers should be mentioned, considering that the use of questionnaires tends to underestimate the degree of work-related stress, professional burnout and job dissatisfaction.

An action plan should be implemented in the hospital management to control the levels of stress, professional burnout and job satisfaction, enhancing the participation and communication between the professionals and the management.

## Conclusions

The professionals reveal average levels of stress and job satisfaction. The level of emotional exhaustion is also average, that of depersonalization is high and that of personal accomplishment low. The factors related to the professional burnout dimensions are: the level of global work-related stress, having trainees under one's supervision, living with relatives and the mean global satisfaction level. The variables related to the stress and job satisfaction level are supervising trainees and age, respectively. Finally, training on self-control and stress management techniques are considered very important to strengthen optimism and self-esteem.
